# Supplementary material for: Exposure–Response Relationship and Doubling Risk Doses—A Systematic Review of Occupational Workload and Osteoarthritis of the Hip
Source: Int J Environ Res Public Health. 2019 Sep 30;16(19):3681. doi: 10.3390/ijerph16193681 (PMC6802007; doi:10.3390/ijerph16193681)
Supplement: Supplementary file 1 [file ijerph-16-03681-s001.zip › File S1.docx]

# **File S1.** The literature search strategy

Our library of studies is based on the comprehensive literature searches done for our meta-analysis in 2019 (Sun et al. 2019)*, which covered the literature published until 2017.

*Sun, Y.; Nold, A.; Glitsch, U.; Bochmann F. Hip Osteoarthritis and Physical Workload: Influence of Study Quality on Risk Estimations—A Meta-Analysis of Epidemiological Findings. Int. J. Environ. Res. Public Health 2019, 16(3). pii: E322. doi: 10.3390/ijerph16030322.

**Search strategy in the meta-analysis published previously by Sun et al. 2019 [5]:**

We searched in PubMed, EMBASE, Cochrane Work and Google Scholar for relevant studies. Details of the search strategy for the meta-analysis are described in the referring supplement file File S1.pdf of that publication [5]. Beforehand, we conducted test searches to improve precision and recall. Tests included synonym controls, check and comparison of MESH terms and terms in EMBASE basic index.

Searches were limited to English and German language articles. Literature with publication date 2010 - 2017 was searched in that meta-analysis. To ensure a completeness of our literature search, we compared the findings of our literature search with the reference lists of published 10 reviews (see list below).

We did not search in Scopus and Web of Science, since Google Scholar (used in our literature search) usually covers nearly all citations in Web of Science and Scopus (see paper of Martin-Martin 2019, https://arxiv.org/ftp/arxiv/papers/1808/1808.05053.pdf).

**Search strategy for this review:**

For this review, we extended the literature search from 2017 to February 2019. Again, we searched in PubMed, EMBASE, Cochrane Work and Google Scholar for relevant studies. We used the same search strategies described above as in Sun et al. 2019 [5]. Furthermore, we checked references in the two new reviews/meta-analysis we found (Gignac et al. 2019, Seidler et al. 2018). Results of update searches see below.

Detailed search strategies used for this review are as follows:

**PubMed:**

14.2.2019 search date

#1 Search "Osteoarthritis, Hip"[Mesh]

#2 Search occupational?

#3 Search lifting or carrying

#4 Search farmer? or farming

#5 Search #2 OR #3 OR #4

#6 Search #1 AND #5

#7 Search ("2017\01\01"[Date - Publication] : "3000"[Date - Publication])

#8 Search #6 AND #7

N hits = 20

**EMBASE:**

18.2.2019 search date, Host STN

L1 S HIP OSTEOARTHRITIS

L2 S COXARTHROSIS

L3 S COXARTHROSES

L4 S L1 OR L2 OR L3

L5 S INDUSTRIAL MEDICINE

L6 S OCCUPATION?

L7 S L5 OR L6

L8 S L4 AND L7

L9 S L8 AND 2010-2017/PY

L10 S L9 NOT THERAPY

L11 S L10 NOT SURGERY

N hits = 13

**Cochrane Work:**

14.2.2019 search date, http://work.cochrane.org/

Hand searching

N hits = 0

**Google Scholar:**

14.2.2019 search date, https://scholar.google.com/

We conducted hand searching with the following terms in different combinations:

Hip osteoarthritis, coxarthrosis, occupation, work, lifting, dose-response, study.

N hits = 3

**Results of update literature seaches:**

We found one new review (Gignac 2019) and one new meta-analysis (Seidler 2018). After comparison with available reference lists, we identified additional four studies which are not relevant for this review.

See also Supplement Table S1.

**Reviews published since 2010 used for reference list check (n=12):**

Allen, K.D.; Golightly, Y.M. State of the evidence. Curr. Opin. Rheumatol. 2015, 27, 276–283.

Bergmann, A.; Bolm-Audorff, U.; Krone, D.; Seidler, A.; Liebers, F.; Haerting, J.; Freiberg, A.; Unverzagt, S. Occupational Strain as a Risk for Hip Osteoarthritis. Dtsch. Arztebl. Int. 2017, 114, 581–588.

Fransen, M.; Agaliotis, M.; Bridgett, L.; Mackey, M.G. Hip and knee pain: Role of occupational factors. Best Pract. Res. Clin. Rheumatol. 2011, 25, 81–101.

Gignac, M.A.M.; Irvin, E.; Cullen, K.; Van Eerd, D.; Beaton, D.E.; Mahood, Q.; McLeod, C.; Backman, C.L. Men and women's occupational activities and the risk of developing osteoarthritis of the knee, hip or hands: A systematic review and recommendations for future research. Arthritis Care Res (Hoboken). 2019 DOI: 10.1002/acr.23855

Harris, E.C.; Coggon, D. Hip osteoarthritis and work. Best Pract. Res. Clin. Rheumatol. 2015, 29, 462–482.

Hartmann, B.; Seidel, D. Koxarthrosen im Erwerbsalter. Eine Übersicht zum aktuellen Erkenntnisstand. [Coxarthrosis during working life. A review]. Arbeitsmed. Sozialmed. Umweltmed. 2011, 46, 670–686.

Johnson, V.L.; Hunter, D.J. The epidemiology of osteoarthritis. Best Pract. Res. Clin. Rheumatol. 2014, 28, 5–15.

Neogi, T.; Zhang, Y. Epidemiology of osteoarthritis. Rheum. Dis. Clin. N. Am. 2013, 39, 1–19.

Richmond, S.A.; Fukuchi, R.K.; Ezzat, A.; Schneider, K.; Schneider, G.; Emery, C.A. Are joint injury, sport activity, physical activity, obesity, or occupational activities predictors for osteoarthritis? A systematic review. J. Orthop. Sports Phys. Ther. 2013, 43, B515–B519.

Seidler, A.; Lüben, L.; Hegewald, J.; Bolm-Audorff, U.; Bergmann, A.; Liebers, F.; Ramdohr, C.; Romero-Starke, K.; Freiberg, A.; Unverzagt, S. Dose-response relationship between cumulative physical workload and osteoarthritis of the hip - a meta-analysis applying an external reference population for exposure assignment. BMC. Musculoskelet. Disord. 2018, 19: 182. DOI: 10.1186/s12891-018-2085-8.

Spahn, G.; Kaiser, M.; Gantz, S.; Schiltenwolf, M.; Hartmann, B.; Schiele, R.; Hofmann, G.O. Systematisches Literatur-Review und Metaanalyse zur Bestimmung von Risikofaktoren für die Koxarthrose [Risk factors for hip osteoarthritis (coxarthrosis). Results from a systematic review and meta-analysis]. Arbeitsmed. Sozialmed. Umweltmed. 2014, 49, 207–222.

Yucesoy, B.; Charles, L.E.; Baker, B.; Burchfiel, C.M. Occupational and genetic risk factors for osteoarthritis: A review. Work 2015, 50, 261–273.
